# First contact, last priority? Reimagining physiotherapy in primary health care

**DOI:** 10.4102/sajp.v81i1.2295

**Published:** 2025-10-08

**Authors:** Witness Mudzi

**Affiliations:** 1 College of Medicine and Health Sciences, University of Rwanda, Kigali, Rwanda; 2 Department of Physiotherapy, Faculty of Health Sciences, University of the Witwatersrand, Johannesburg, South Africa

## Introduction

South Africa’s evolving health landscape, driven by the National Health Insurance (NHI) initiative, demands a radical rethinking of professional roles within primary health care (PHC). Implementing the NHI scheme promises universal health coverage (UHC), aiming to dismantle historical inequities and ensure equitable access to care. As the country grapples with a quadruple burden of disease, human immunodeficiency virus or acquired immune deficiency syndrome (HIV/AIDS), non-communicable diseases, maternal and child mortality and violence-related injuries, the need for accessible, community-based care has never been more urgent (Myezwa & Van Niekerk 2013). Within this context, physiotherapists are uniquely positioned to serve as autonomous (first-line) practitioners, especially in underserved areas with limited access to physicians (Diener, 2008). However, their integration into the PHC system remains limited and uneven. Within this reform, physiotherapists, long confined to secondary and tertiary care, are poised to redefine their role as first-contact practitioners in PHC. This editorial examines the systemic context, workforce realities, educational gaps and opportunities for physiotherapists to serve as first-contact practitioners across the life course and in diverse clinical domains at the PHC level.

## National Health Insurance: A new framework for equity

The *NHI Act 20 2023* aims to establish a single-payer system designed to provide comprehensive health services to all South Africans, regardless of socioeconomic status. It encompasses promotive, preventive, curative, rehabilitative and palliative care, aligning with global UHC goals and South Africa’s constitutional mandate to ensure access to health care as enshrined in Section 27(1) of the constitution (Constitution of the Republic of South Africa 1996).

However, rehabilitation services, particularly physiotherapy, remain underrepresented in NHI pilot programmes and strategic documents (Myezwa & Van Niekerk 2013). This oversight risks excluding a profession that could significantly reduce hospital admissions and improve functional outcomes at the community level (Narain & Mathye 2025).

Physiotherapists can play a vital role in the early detection and management of various medical conditions, which are among the leading contributors to disability-adjusted life years (DALYs) in South Africa (Narain & Mathye 2023). Their integration into PHC aligns with the World Health Organization’s (WHO) call for multidisciplinary teams to achieve UHC.

## The role of National Health Insurance and primary health care re-engineering

The NHI Bill presents both a challenge and an opportunity. Section 7(2)(b) of the bill mandates that access to all health services, including physiotherapy, should occur at the PHC level. However, without deliberate planning, physiotherapy risks being excluded from implementation. Physiotherapy must be part of this vision, not as an afterthought, but as a cornerstone of preventive and rehabilitative care.

### Physiotherapy workforce in South Africa: A landscape of inequity

As of March 2025, South Africa had 9235 registered physiotherapists for ~ 64 million people (≈1:6930), with the vast majority working in the private sector (Health Professions Council of South Africa [HPCSA] 2025). In public rural facilities, ratios fall to fewer than 1 physiotherapist per 10 000 uninsured people (Rule, Lorenzo & Wolmarans 2022). Rehabilitation workforce density is under 0.5 per 10 000 people, far below the WHO’s indicative target of 2.5 per 10 000 (WHO 2017). This imbalance leaves vast populations, especially in rural areas, without first-contact rehabilitation services (Conradie, Berner & Louw 2022; Louw & Uys 2014; Rule et al. 2022).

Despite their potential, physiotherapists are unevenly distributed across the health system. Only 3% work in primary care settings, while 80% are concentrated in secondary and tertiary hospitals (Conradie et al. 2022). The urban-rural divide is stark: rural provinces such as Limpopo and Eastern Cape have fewer than 1 physiotherapist per 10 000 people (Conradie et al. 2022). Moreover, the public–private imbalance exacerbates inequity. According to Naidoo (2012), 84% of South Africans rely on public health services, yet most physiotherapists work in urban private practices, thus serving only 16% of the population. This leaves public sector facilities critically understaffed, especially in rural areas (Conradie et al. 2022). Bridging this gap is not just a policy issue; it’s a matter of social justice.

Workforce development is further hampered by limited postgraduate training and a lack of incentives for rural deployment (Narain & Mathye 2025). Without strategic investment, the profession risks being sidelined in the very system it could help transform.

Physiotherapy’s exclusion from PHC disproportionately affects marginalised populations. The Constitution of South Africa guarantees the right to access health care services. Realising this right requires a health system that includes rehabilitation as a core component, not a luxury.

### The case for physiotherapists in primary health care

Physiotherapists possess a unique blend of diagnostic acumen, rehabilitative expertise and preventive insight. Their ability to manage musculoskeletal conditions, chronic diseases of lifestyle, cardiorespiratory conditions and promote functional independence makes them ideal candidates for PHC integration. In rural and underserved communities, where access to physicians may be limited, physiotherapists can offer timely interventions that reduce the burden on hospitals and improve patient outcomes (Narain & Mathye 2023).

### Barriers to primary health care integration and first-contact role

Despite their potential, several systemic challenges hinder physiotherapists from assuming a first-contact role:

#### Policy marginalisation

Physiotherapy is often omitted from key health policy documents, limiting its visibility and strategic inclusion in PHC frameworks. The *NHI Act 20 of 2023* does not explicitly integrate physiotherapists into PHC referral structures, which can lead to underutilisation and compromised health care service delivery.

#### Workforce distribution

The profession suffers from an urban-centric workforce, with rural areas facing acute shortages. The public sector suffers from a severe shortage of physiotherapists, especially in rural areas. Most physiotherapists are in the private sector, leaving PHC clinics understaffed (Rule et al. 2022).

#### Professional hierarchies

A rigid medical hierarchy often relegates physiotherapists to subordinate roles, undermining their autonomy and scope of practice. Doctors and nurses usually dominate PHC decision-making, marginalising rehabilitation professionals (Mlenzana et al. 2013).

#### Education gaps

Current training models emphasise curative care over health promotion and disease prevention, misaligning with PHC principles. Curricula remain hospital- and impairment-focused, underpreparing graduates for PHC and preventive care roles (M’kumbuzi & Myezwa 2016).

#### Public awareness

Many South Africans are unaware of what physiotherapists do, leading to underutilisation of their services. Many patients are unaware that they can consult physiotherapists directly, and some medical aids still require a general practitioner (GP) referral (Hanass-Hancock et al. 2017).

### First-contact status: Legal and professional recognition

Physiotherapists in South Africa have held autonomous (first-line) practitioner status since 1985, formally recognised by the HPCSA in 1997 (South African Society of Physiotherapy [SASP] 2016). This designation allows them to:

- Diagnose and treat patients autonomously
- Refer for imaging or specialist care
- Issue medical certificates and manage chronic conditions

Physiotherapists do not require a referral to see a patient. Yet, despite this legal recognition, physiotherapists are often perceived as secondary providers, consulted only after physician referral (Narain, Mathye & Mtshali 2023). This perception must shift if South Africa is to fully leverage its rehabilitation workforce under the NHI.

### Strategic recommendations

To reposition physiotherapists as first-contact providers playing a prominent PHC-level role under the NHI, South Africa must focus on the following aspects:

#### Policy representation

Physiotherapists must be actively involved in health policy development. Narain et al. (2023) note that their limited policymaking role stems from weak bargaining power and poor interprofessional communication. Physiotherapy must be embedded in NHI service packages, ensuring funding and representation in policy frameworks (Narain & Mathye 2025; Narain et al. 2023). Strengthening professional associations and fostering collaboration with government bodies can help rectify this.

#### Workforce expansion

The government must invest in expanding the physiotherapy workforce, particularly in rural and underserved areas. Incentives such as scholarships, housing allowances and career development opportunities can attract professionals to these regions (Conradie et al. 2022). The frozen physiotherapy posts in public hospitals should be revisited.

#### Interprofessional collaboration

In integrated teams, physiotherapists should work alongside nurses, doctors and community health workers. This requires dismantling professional hierarchies and fostering mutual respect across disciplines.

#### Evidence-based practice

Robust research is needed to demonstrate physiotherapy’s cost-effectiveness and health impact in PHC. Studies should evaluate outcomes such as reduced hospital admissions, improved functional status and enhanced quality of life.

#### Reform physiotherapy education

Universities should revise physiotherapy curricula to include modules on PHC, public health and health systems management if not in place or strengthened where they are in place. Exposure to community-based practice during training can prepare graduates for frontline roles. Curricula must emphasise community health, prevention and interdisciplinary collaboration (Myezwa & Van Niekerk 2013).

#### Launch public awareness campaigns and professional advocacy

Running campaigns promoting physiotherapy as a first-contact option is important. Improving societal knowledge of physiotherapy is essential. Campaigns should highlight the profession’s role in managing chronic conditions, promoting mobility and function and preventing disability. Strengthening professional advocacy through bodies such as the SASP is also important.

### A vision for the future

Imagine a South African clinic where a patient with early signs of arthritis walks in and is immediately assessed by a physiotherapist, and no referral is needed. The therapist not only provides targeted interventions but also educates the patient on lifestyle modifications, preventing disease progression. This is not a distant dream but a tangible future, contingent on systemic reform and visionary leadership.

## Conclusion

Physiotherapists in South Africa have the scope, competencies and legal authority to serve as first-contact practitioners. The underutilisation of this workforce represents a missed opportunity within the national reform agenda. For NHI to achieve equitable access, physiotherapists must be integrated into PHC as first-contact providers, supported by workforce redistribution, education reform and policy recognition. As South Africa rolls out NHI, embracing physiotherapy as a cornerstone of PHC will be vital to achieving health equity, system sustainability, efficiency and excellence in health care.
